# Snack and Nutrient Intake Status of Top-Level Female University Athletes: A Cross-Sectional Study

**DOI:** 10.3390/healthcare12040468

**Published:** 2024-02-13

**Authors:** Hiromi Inaba, Fumi Hoshino, Mutsuaki Edama, Go Omori

**Affiliations:** 1 Athlete Support Research Center, Niigata University of Health and Welfare, 1398 Shimami-cho, Kita-ku, Niigata 950-3198, Japan; fumi-h@nuhw.ac.jp (F.H.); edama@nuhw.ac.jp (M.E.); omori@nuhw.ac.jp (G.O.); 2 Department of Health and Nutrition, Faculty of Health Science, Niigata University of Health and Welfare, Niigata 950-3198, Japan; 3 Institute for Human Movement and Medical Sciences, Niigata University of Health and Welfare, Niigata 950-3198, Japan; 4 Department of Health and Sports, Niigata University of Health and Welfare, Niigata 950-3198, Japan

**Keywords:** female athlete, snack, energy intake, sports nutrition, dietary supplement

## Abstract

Ensuring proper energy, nutrient intake, and sleep is vital for athlete health and competitiveness. Despite previous studies investigating the nutrient intake among top-level collegiate female athletes in Japan, the status of snack consumption remains unclear. This study addressed this gap by surveying 70 top-level female university athletes. The survey included a self-administered diet history questionnaire, a qualitative food intake frequency survey, and a survey on snack and dietary supplement use. The results revealed a low frequency of snack intake (2.1 ± 2.3 days/week), with 55.7% of athletes reporting snack consumption. The energy intake in the snack-intake group was significantly higher than that in the without-snack-intake group (31.5 ± 10.0 vs. 26.6 ± 9.92 kcal/kg of BM, *p* = 0.047). Similarly, carbohydrate intake was significantly higher in the snack-intake group than in the without-snack-intake group (4.84 ± 1.71 vs. 3.96 ± 1.65 g/kg of BM/day, *p* = 0.035). However, neither group reached the recommended value of 5–8 g/kg of BM/day during the medium training period. Overall, this study emphasizes inadequate energy intake even among athletes with a high snack intake frequency, highlighting the necessity to enhance overall food consumption and underscoring the importance of nutritional education for incorporating appropriate complementary meals to improve performance.

## 1. Introduction

In addition to proper practice, nutrition, and sleep, athletes must prioritize their health to sustain continuous competition [1]. Diet forms the foundation of an effective training program aimed at achieving optimal enhancement, with particular emphasis on adequate carbohydrate intake as a crucial energy source [2]. The recommended amount of carbohydrates varies with exercise intensity, ranging from 3–5, 5–7, 6–10, to 8–12 g/kg of body mass for low, moderate, high, and very high levels of intensity, respectively [2]. In endurance sports, performance is intricately associated with the availability of carbohydrates as an energy substrate, making sufficient carbohydrate intake imperative [3]. Researchers have previously asserted that consuming carbohydrates before exercise enhances performance. Additionally, as a standard practice, nutrition experts recommend the consumption of 800–1200 kcal of carbohydrates (low in dietary fiber) 2–3 h before the commencement of practice or sports competition [4,5,6].

Ideally, a pre-practice or pre-sports competition supplemental meal (snack) should include carbohydrates, a moderate amount of protein, and low levels of fat and fiber to avoid delaying stomach emptying [7]. Consuming carbohydrates immediately after exercising is an effective strategy for enhancing glycogen resynthesis and storage, maximizing the activation of glycogen synthase [8]. A mere 2 h delay in carbohydrate intake can significantly decrease the rate of glycogen synthesis [9]. Hence, snacks high in carbohydrates are recommended. Although muscle tissue degradation increases after training or transient exercise, previous studies have shown that consuming a meal immediately following a game can enhance muscle protein synthesis and recovery in athletes [10]. Researchers have also emphasized that the rate of muscle protein synthesis is approximately three times higher when a meal containing high-quality protein and carbohydrates is consumed within 1 h after exercise compared with consuming the same meal composition 3 h after exercise [11].

Meeting the necessary carbohydrate intake from the three main meals (i.e., breakfast, lunch, and dinner) poses a challenge for athletes. Consequently, a common practice is to supplement carbohydrates through snacks consumed before, after, or during practice, as well as before breakfast or after dinner. This approach is crucial for the rapid recovery of glycogen in the muscles [12].

Fat intake recommendations from the Institute of Medicine suggest that healthy women should consume no more than 20%–35% of their total energy intake from fat, with no more than 10% of their energy intake coming from saturated fatty acids [13]. This lipid intake is approximately 1.0 g per kg of body mass, but it may be higher depending on fitness level and energy needs. For instance, it has been reported that endurance athletes may require a fat intake of 2.0 g per kg of body mass to meet their energy requirements as these athletes often possess an increased metabolic capacity to utilize lipids as an energy source [14]. In the Dietary Reference Intakes for Japanese (2020 edition), the recommended amount of lipids is 20%–30% of the total energy intake [15].

Avoidance of the female athlete triad (FAT) is crucial for female athletes to protect their health during competition. Insufficient energy intake can lead to short-term weight loss and, in the long term, elevate the risk of FAT. The American College of Sports Medicine defines FAT as the occurrence of low energy availability (LEA) with or without eating disorders, hypothalamic amenorrhea, and osteoporosis [16]. In a study of Japanese female university athletes, Sawai et al. reported that energy deficiency induces FAT [17]. Additionally, several studies on FAT have been conducted on Japanese collegiate female athletes [18,19]. Edama et al. explored the relationship between FAT and the frequency of injury [20]. Moreover, LEA increases the risk of amenorrhea [16,21]. Therefore, maintaining an adequate daily energy intake is crucial for female athletes to lead a healthy athletic life and sustain good health after retiring from sports competitions.

College athletes are not only dedicated to their sport but also juggling schoolwork, part-time jobs, and, if they are living alone, general household chores. Balancing all these activities and ensuring that they obtain the necessary amount of energy can be challenging [22]. However, studies documenting the frequency and content of snack intake by female collegiate athletes are scarce, making it difficult to comprehend the current status of snack consumption in this population. Moreover, only few researchers have reported on the frequency of snack intake among Japanese female college athletes. Therefore, the present study aimed to specifically focus on Japanese college students.

The objective of the study was to determine the snack, energy, and nutrient intakes of female college athletes in Japan. We hypothesized that top-level college female athletes in Japan have high levels of energy and carbohydrate intake when consuming snacks more frequently.

## 2. Materials

### 2.1. Participants and Survey Period

A total of 70 female university students belonging to clubs designated for training (e.g., volleyball, basketball, soccer, swimming, and track and field) were recruited to undergo a survey from December 2022 to February 2023. The surveys were conducted during the training period. Each of the clubs was at the national tournament level in Japan. All female athletes were informed of the procedures, benefits, and risks of their involvement in the study and provided written informed consent prior to participation. Additionally, they were informed that participation was voluntary and that they could withdraw at any time.

### 2.2. Ethical Considerations

This study was conducted in accordance with the Declaration of Helsinki [23] and the Ethical Guidelines for Medical Research Involving Human Subjects (Ministry of Education, Culture, Sports, Science and Technology and Ministry of Health, Labour and Welfare 2017) [24]. The study was approved by the institutional review board of Niigata University of Health and Welfare (approval number: 18831-220526).

### 2.3. Methods and Dietary Survey

The height, body mass, and body composition of the participants were measured using a digital height meter (AD6400, A&D, Tokyo, Japan) or InBody470 (Inbody Japan, Tokyo, Japan). A survey on the frequency of food intake and another on the frequency of qualitative food intake were conducted on the same day using the Brief Self-Administered Diet History Questionnaire (BDHQ) [25,26].

The BDHQ is a four-page, fixed-portion questionnaire that inquires about the frequency of consuming selected foods (without specifying portion size) to estimate the dietary intake of 58 food and beverage items over the preceding month [27]. The BDHQ consists of five sections as follows: (1) frequency of intake of food and nonalcoholic beverage items, (2) daily intake of rice and miso soup, (3) frequency of drinking and amount per drink for alcoholic beverages, (4) typical cooking methods, and (5) general dietary behavior [24]. Respondents reflect on these items for the previous month. Owing to the large weight estimation error associated with carbohydrates [22], the study, for rice intake, presented food models of four bowl sizes and rice balls to provide an accurate depiction of the amount of rice consumed by the participants. Additionally, the survey included questions about the frequency of consuming snacks, the types of snacks consumed, and the timing of consumption. These questions were administered through Google Forms. Furthermore, a survey on the use of dietary supplements, including product names and the timing and method of consumption, was conducted. For participants confirming themselves as dietary supplement users, supplementation was added to the BDHQ results for energy and nutrient intake.

The survey on the frequency of qualitative food intake required the participants to respond to 12 questions about their weekly food consumption habits (with the highest frequency being 7 days). The items were as follows: (1) number of days refraining from consuming sweets and soft drinks (e.g., juice), (2) number of days consuming three meals (breakfast, lunch, and dinner), (3) number of days prioritizing consuming staple foods (e.g., rice, bread, and noodles), (4) number of days consuming eggs or egg dishes, (5) number of days consuming meat and fish side dishes, (6) number of days consuming soybean products (e.g., tofu, and natto), (7) number of days with green and yellow vegetables (e.g., carrots, spinach, and pumpkin), (8) number of days consuming light-colored vegetables (e.g., cucumber, cabbage, and lettuce), (9) number of days consuming milk/yogurt, (10) number of days consuming fruit (including 100% juice), (11) number of days consuming breakfast, and (12) number of days consuming snacks.

The staff, including managers, coaches, and trainers, were not allowed to be present during the survey. However, two registered dietitians were present during the survey.

### 2.4. Statistical Analysis

Data were analyzed using R (version 4.1.0.) [28]. A *t*-test was used for comparisons between the following two groups: the snack-intake group and the without-snack-intake group. The former comprised individuals who reported consuming a snack at least one day a week, whereas the latter comprised those who did not consume a snack at least one day a week. For the comparison of proportions, Fisher’s exact probability test was used. The significance level was set to *p* < 0.05.

## 3. Results

**Figure 1:**
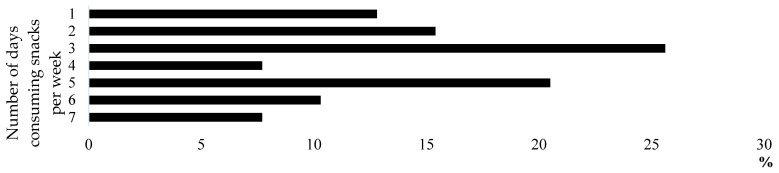
Percentage of snack intake days among athletes who were habitual snackers.

Table 1 presents the physical characteristics of the participants. We found no differences in body mass between the snack-intake and without-snack-intake groups. Thirty-nine athletes (55.7%) habitually consumed snacks (at least one day per week), and their mean (±SD) frequency of snack intake was 3.7 ± 1.8 days/week (Figure 1). Regarding the clubs to which the athletes belonged, the basketball club had the highest number of athletes (*n* = 20), followed by volleyball (*n* = 16), swimming (*n* = 13), soccer (*n* = 12), and track and field (*n* = 9; Table 2).

**Figure 2:**
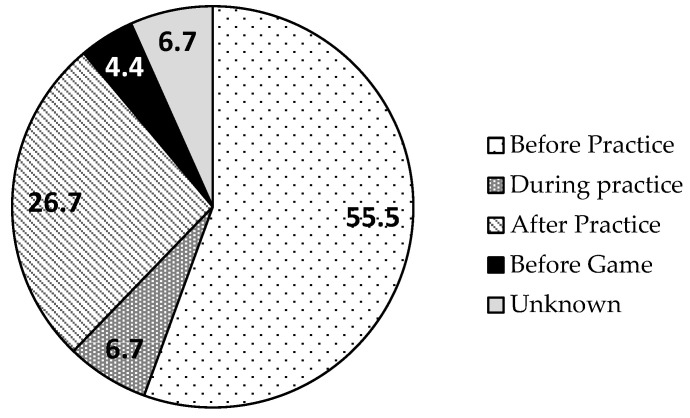
Percentages of snack times for female athletes.

Figure 2 presents the timing of snack intake. The most common time for snacking was before practice (55.6%). The energy intake of the snack-intake group was significantly higher than that of the without-snack-intake group (1844 ± 557 vs. 1522 ± 504 kcal, *p* = 0.015). Protein, fat, and carbohydrate intakes for all the participants were 58.4 ± 21.5, 44.3 ± 16.2, and 257.0 ± 94.6 g/day, respectively (n = 70). Comparing the two groups in terms of intake per kilogram of body mass, energy (*p* = 0.047), carbohydrate (*p* = 0.035), and vitamin C (*p* = 0.047) intakes were significantly higher in the snack-intake group than in the without-snack-intake group (Table 3).

Table 4 shows the percentages of nutritional intake in the snack-intake and without-snack-intake groups relative to the dietary intake standards for Japanese people; no significant difference was observed between the two groups. The percentages of athletes who exceeded the recommended percentage in the fat–energy ratio were 12.9% (without-snack-intake group) and 3.2% (with-snack-intake group), respectively. Additionally, the percentage of athletes whose protein–energy ratio was lower than the recommended ratio was 41.9% in the without-snack-intake group and 48.7% in the with-snack-intake group.

Table 5 presents the results of the survey on the frequency of qualitative food intake. Soy products, such as tofu, natto, and atuage (tofu fried in oil; *p* = 0.084), and fruit (including 100% fruit juice, *p* = 0.072) tended to be consumed more frequently by the snack-intake group than by the without-snack-intake group.

Table 6 presents the specific types of snacks that were consumed. The most common food was rice balls (36.7%), followed by bread and energy gels. Steamed chicken, ranked fourth, refers to tender steamed chicken breast; it is high in protein and low in fat and is popular among athletes as a readily available option at convenience stores, supermarkets, and drugstores. Table 7 presents the types of dietary supplements taken by the participants, with dietary protein being the most common followed by vitamins and amino acids.

Table 7 presents the dietary supplements that the participants used (multiple answers possible). A total of 22 participants (31.4%) were using dietary supplements. We found no significant difference in dietary supplement intake between the two groups as follows: 13 (33.3%) in the snack-intake group and 9 (29.0%) in the without-snack-intake group (*p* = 0.798, Fisher’s exact test). Protein was the most frequently consumed nutrient (n = 11). Three respondents (4.3%) used more than one supplement.

## 4. Discussion

This study aimed to determine the frequency of snack intake as well as the nutrient and overall intake status of female college athletes, providing a basis for future nutritional support. The study revealed a low frequency of snack intake, with only 39 athletes (55.7%) habitually consuming snacks (at least once a week), averaging 3.7 days per week. Athletes with more frequent snack consumption exhibited significantly higher energy intake. Snacks were predominantly consumed before and after practice (82.3%), and 22 female athletes (31.4%) reported using dietary supplements.

Consuming snacks is crucial for athletes to obtain the energy and nutrients that may not be fully acquired during the three main meals, ensuring optimal health and sports performance [29]. However, only 55.7% of the athletes had the habit of snacking, and even among those who did, the frequency was low, averaging 3.7 days per week. In a study of female elite handball players in Japan, Suzuki et al. [30] found that complementary meals were consumed thrice daily, including morning, afternoon, and snacks before sleeping. These athletes consumed at least one snack daily. In a study on NCAA Division I female college athletes in the United States, the mean frequency of snack intake was 2.2 ± 1.2 times per day, correlating with that of carbohydrate intake [31]. Burke et al. [32] reported in a study on Australian Olympians that the average frequency of snack intake was twice daily, constituting 23% of daily energy intake. In our study, the energy intake difference between the snack-intake (1844 ± 557 kcal) and without-snack-intake (1522 ± 504 kcal) groups was approximately 322 kcal. Using this value, the energy intake from supplemental food was calculated to be 17.4%, which was considerably lower than the value reported by Burke et al. The overall energy intake of 1844 ± 557 kcal for the snack-intake group was also low, emphasizing the importance of not only increasing energy consumption during main meals but also providing guidance on obtaining energy from snacks that may be missed during these meals.

In a study on elite Canadian athletes, Erdman et al. [33] reported that snacks were consumed 57% of the time in the morning, 71.6% in the afternoon, and 58.1% in the evening, with 24.3% of total energy intake coming from snacks. The number of athletes (n = 6; 8.6%) in the present study who consumed snacks daily on practice days was lower than that in previous studies, suggesting that the overall energy and nutrient intake of the participants was inadequate.

The intake of carbohydrates and vitamin C were significantly higher in the snack-intake group than in the without-snack-intake group (Table 3). The International Society of Sports Nutrition recommends that if the rapid restoration of glycogen levels is required (<4 h of recovery time), then a combination of carbohydrates (0.8 g/kg/h) and protein (0.2–0.4 g/kg/h) is required [29]. The current study infers that the intake of protein-rich foods is required in this group of participants. We also consider that the intake of vitamin C was significantly higher in the snack-intake group owing to the higher consumption of fruits, including 100% fruit juice (Table 5).

Table 4 presents the percentage of athletes meeting the Dietary Reference Intakes Japanese for various nutrients, with no difference in the compliance rate between the snack-intake and without-snack-intake groups. It is noteworthy that many nutrients did not meet the standard. However, in a study conducted by Kobayashi et al. on 4017 female university students, the sufficiency rate of various nutrients was low, and the results of this study support the findings of Kobayashi et al. [34]. The percentage of athletes whose fat energy percentage was between 20 and 30% of their total energy was 74.2% in the without-snack-intake group and 64.1% in the with-snack-intake group. The percentages of athletes who exceeded the recommended percentage were 12.9% and 3.2%, respectively, and the higher percentage of the without-snack-intake group had higher fat energy percentages than in the with-snack-intake group, which was a cause for concern. Additionally, the percentage of athletes whose protein–energy ratio was lower than the recommended ratio was 41.9% in the without-snack-intake group and 48.7% in the with-snack-intake group. The intake balance of macronutrients needs to be closer to the recommended ratio.

There were no significant differences in carbohydrate intake between the athletes who consumed dietary supplements (4.59 ± 1.81 g/kg of BM) and those who did not (4.36 ± 1.57 g/kg of BM). Even in the supplement intake group, carbohydrate intake did not reach the recommended amount of 5–8 g/kg of BM/day (moderate training period) as suggested by Burke et al. [2]. We propose that consuming sufficient carbohydrates from diet is important for athletes to increase muscle glycogen storage for performance enhancement, conditioning maintenance, and injury prevention [35]. Moreover, supplementing carbohydrates from snacks is appropriate but insufficient. The American College of Sports Medicine recommends consuming 1.0–1.2 g/kg of BW of carbohydrates every hour from the end of training to the start of the next training session when multiple sessions occur in a day to restore depleted muscle glycogen levels [36]. In the future, it is essential to elucidate the reasons for low levels of snack intake and to reiterate nutritional education on the importance of such intake.

Eating habits and patterns are crucial for athletic performance and recovery as they influence energy expenditure and nutrient intake [37]. Nutrition experts recommend consuming foods rich in carbohydrates and protein, such as 100% fruit juice, rice balls, sandwiches, and milk, as a complementary meal early after exercise. However, the current study could not specify the names of specific dishes and foods. Nevertheless, soy products such as tofu and natto (*p* = 0.084) and fruits (*p* = 0.072) tended to be consumed more frequently in the snack intake group than in the without-snack-intake group (Table 5). The study suggests the necessity of nutritional guidance concerning both the quality and frequency of snack intake for rapid muscle glycogen recovery, performance enhancement, and injury prevention. For instance, LEA can lead to amenorrhea and osteoporosis [16,21], with the risk increasing below 30 kcal/kg of lean body mass/day [38]. Previous studies have reported inadequate energy and nutrient intakes in many athletes [39]. Athletes without snack intake habits may experience more pronounced inadequate energy intake, leading to LEA. Thus, the study recommends nutritional support aimed at increasing energy intake.

Protein is essential for supporting recovery and adaptation after exercise training, especially in sports such as soccer that induce substantial musculoskeletal stress, disruption, and damage [40]. To aid recovery and adaptation, dietary protein is necessary [41]. Major sports nutrition organizations advocate that athletes aiming to optimize body composition and sports performance should consume 1.2–2.0 g protein/kg of BW/day, with high intake potentially advantageous under certain conditions [8,39]. In the current study, the without-snack-intake group had a protein intake of 0.97 g/kg of BM/day, whereas the snack-intake group had a protein intake of 1.05 g/kg of BM/day, which were both below the recommended protein amount. Among the six participants using protein as a dietary supplement, all but one consumed more than 1.4 g/kg of BM/day. Therefore, in addition to carbohydrate intake, education on protein intake is crucial.

The total fat intake was 44.3 ± 16.2 g/day, with an energy ratio of 23.4 ± 8.6%, which is within the recommended range of 20%–30% according to the Dietary Reference Intakes for Japanese (2020 edition). This ratio was deemed appropriate as an energy ratio [15]. Considering concerns about energy deficits in both the snack-intake and without-snack-intake groups, there is a need to increase fat intake in addition to carbohydrate and protein intakes.

The rate of dietary supplement use was 31.4% (n = 22, Table 7). Tabata et al. [42] conducted a study on 574 Japanese male and female track and field athletes (including 275 junior athletes [aged <20 years] and 299 senior athletes) who competed in international competitions between 2013 and 2018. The authors reported a dietary supplement use rate of 58.9% for junior athletes and 69.8% for senior athletes. Although the participants in the present study were top-level collegiate athletes, their use of dietary supplements was found to be lower than that of participants in the study conducted by Tabata et al. [42]. Among the current study participants, nine were track and field athletes of whom four (44.4%) were dietary supplement users. This value is low when specifically compared with track and field athletes. Knapik et al. [43] conducted a systematic review and meta-analysis and found that elite athletes used dietary supplements more frequently than their non-elite counterparts. These subjects may have lower levels of awareness regarding food than track and field athletes participating in international competitions. The use of dietary supplements in track and field athletes has been reported to be high [44,45]; therefore, it is possible that dietary supplement use was low when multiple sports were included, similarly to the case of the current study.

In a 2020 survey on supplement use among Japanese high school students, it was found that the supplements used by male and female students differed. Among female students, the most used supplement was vitamin C, followed by protein and iron [46]. Additionally, in a 2006 survey on university students, the most used supplement was amino acids, followed by iron and vitamin C [47]. Therefore, we infer that the types of supplements used in the present study differed depending on the target age group and survey period.

In the present study, the nutrients with the highest intake through dietary supplements were protein (15.7%), vitamins (10.0%), and amino acids (5.7%). In a study on adolescent athletes (18 sports) from four countries (Japan, Germany, Serbia, and Croatia), the highest rate of protein intake through dietary supplements was reported at 55.7 [48]. Although the protein intake rate in the present study was lower (15.7%), it was similar to that in the aforementioned previous study in that protein was the most used dietary supplement. Tabata et al. reported that the most commonly consumed ingredient for Japanese track and field athletes was amino acids (49.3%), followed by vitamins (48.3%), minerals (22.8%), and protein (17.8%). However, the results of the present study do not align with those reported by Tabata et al.; we attribute this difference to the fact that the current study included a wide variety of sports, including basketball, volleyball, and soccer, in addition to track and field.

This study had some limitations. First, it was conducted in a single university in a rural city; therefore, we were unable to adequately consider the area of residence, the season of the study period, and intraindividual variation. Second, we only surveyed 70 female athletes; therefore, the reproducibility of the results could not be verified. Third, we did not examine FAT scores; therefore, we could not confirm whether the female athletes were currently experiencing FAT. We believe that further investigation is necessary. Fourth, although all the self-reported dietary survey methods are subject to reporting errors [49,50,51], especially for athletes [52,53], accounting for large effects of reporting errors is necessary. Fifth, the survey was conducted during the training season; therefore, conducting surveys during other seasons, such as that of the competition season, is also necessary. Sixth, we only conducted a simple two-group comparison. In the future, we need to secure a higher number of participants and conduct a binomial logistic analysis or GLM models with snack consumption habits as the objective variable. Using the results of this study as a basis, we intend to examine nutritional support methods for athletes in the future.

## 5. Conclusions

The frequency of snack intake among college female athletes was low. Moreover, even among athletes with more frequent consumption of snacks, the majority of snacks consisted of confectionery and favorite beverages. Thus, we infer that nutritional guidance on appropriate complementary foods is necessary for improving athletic performance. The results of this study are expected to provide useful information for athletes and nutrition staff who provide nutritional support to athletes.

## Figures and Tables

**Table 1:** Physical characteristics and *p*-values of female athletes according to frequency of snack intake.

		Total	Snack-Intake Group	Without-Snack-Intake Group	*p*-Value
		n = 70	n = 31	n = 39	
Age	(year)	20 ± 1	20 ± 1	20 ± 1	0.456
Height	(cm)	164.3 ± 5.8	163.8 ± 5.8	164.7 ± 5.8	0.511
Body mass	(kg)	58.8 ± 6.5	58.2 ± 6.4	59.2 ± 6.7	0.513
BMI	(kg/m^2^)	21.8 ± 1.8	21.7 ± 1.9	21.8 ± 1.7	0.784
Body fat	(%)	22.8 ± 4.5	22.6 ± 4.8	22.8 ± 4.1	0.852
LBM	(kg)	45.3 ± 4.1	44.8 ± 3.4	45.6 ± 4.6	0.483

Data are presented as means ± SD. BMI, body mass index; LBM, lean body mass.

**Table 2:** Club activities of female athletes, number, and percentages.

Club	n	%
Basketball	20	28.6
Volleyball	16	22.9
Swimming	13	18.6
Soccer	12	17.1
Track and field	9	12.9
Total	70	100.0

**Table 3:** Mean and standard deviation of energy and nutrient intakes by frequency of snack intake.

Energy or Nutrients		Total	Without-Snack-Intake Group	Snack-Intake Group	*p*-Value
		n = 70	n = 31	n = 39	
Energy	(kcal/kg of BM)	29.3 ± 10.3	26.6 ± 9.92	31.5 ± 10.00	0.047
Protein	(g/kg of BM)	1.01 ± 0.40	0.97 ± 0.43	1.05 ± 0.37	0.396
Fat	(g/kg of BM)	0.76 ± 0.29	0.70 ± 0.30	0.80 ± 0.27	0.197
Carbohydrates	(g/kg of BM)	4.45 ± 1.76	3.96 ± 1.65	4.84 ± 1.71	0.035
Total fiber	(g/kg of BM)	0.17 ± 0.08	0.14 ± 0.07	0.17 ± 0.08	0.231
Vitamin B1	(μg/kg of BM)	7.95 ± 9.81	6.50 ± 4.94	9.10 ± 3.49	0.236
Vitamin B2	(μg/kg of BM)	11.9 ± 12.60	10.2 ± 8.57	13.3 ± 12.2	0.283
Vitamin B6	(μg/kg of BM)	11.7 ± 14.60	10.1 ± 8.65	13.0 ± 14.8	0.381
Vitamin B12	(ng/kg of BM)	59.5 ± 72.9	60.2 ± 87.6	54.1 ± 17.8	0.516
Vitamin C	(mg/kg of BM)	1.99 ± 2.66	1.35 ± 6.99	2.51 ± 56.9	0.047
Retinol activity equivalent	(μg RAE/kg of BM)	7.91 ± 6.81	6.52 ± 4.23	9.00 ± 8.06	0.107
Vitamin D	(μg/kg of BM)	0.14 ± 0.12	0.16 ± 0.50	0.13 ± 0.09	0.255
Alpha-tocopherol	(mg/kg of BM)	0.11 ± 0.07	0.10 ± 0.07	0.12 ± 0.06	0.504
Vitamin K	(μg/kg of BM)	5.12 ± 3.19	4.65 ± 2.62	5.49 ± 3.49	0.257
Na	(mg/kg of BM)	56.5 ± 16.2	53.9 ± 17.1	58.6 ± 14.8	0.244
K	(mg/kg of BM)	31.0 ± 14.4	27.8 ± 13.2	33.6 ± 4.6	0.090
Ca	(mg/kg of BM)	6.50 ± 3.10	5.83 ± 3.20	7.00 ± 2.89	0.138
Mg	(mg/kg of BM)	3.20 ± 1.30	2.94 ± 1.30	3.42 ± 1.30	0.122
P	(mg/kg of BM)	13.9 ± 5.34	13.0 ± 5.80	14.5 ± 4.75	0.268
Fe	(mg/kg of BM)	0.11 ± 0.06	0.11 ± 0.05	0.13 ± 0.06	0.324

BM, body mass; RAE, retinol activity equivalent. The numbers indicate the amount of energy or nutrients per kilogram of body mass.

**Table 4:** Dietary Intake Reference for Japanese values (2020 edition, DRIs) and the percentage of athletes who met the values of DRIs.

Nutrients	Reference Value		Without-Snack-Intake Group	Snack-Intake Group	*p*-Value
			n = 31	n = 39	
Protein	E%	13–20	51.6	48.7	0.809
Fat	E%	20–30	74.2	64.1	0.366
Carbohydrates	E%	50–65	61.3	56.4	0.681
Vitamin B1	EAR	0.9	22.6	20.5	0.834
Vitamin B2	EAR	1.0	41.9	53.8	0.322
Vitamin B6	EAR	1.0	35.5	38.5	0.798
Vitamin B12	EAR	2.0	90.3	89.7	0.936
Vitamin C	EAR	85	35.5	53.8	0.126
Retinol equivalent	AI	650	12.9	20.5	0.140
Vitamin D	AI	8.5	45.2	30.8	0.401
Alpha-tocopherol	AI	6.0	22.6	53.8	0.216
Vitamin K	AI	150	67.7	84.6	0.064
K	AI	2600	25.8	41.0	0.183
Ca	EAR	550	9.7	20.5	0.216
Mg	EAR	230	16.1	28.2	0.232
P	AI	800	38.7	53.8	0.208
Fe ^(a)^	EAR	8.5	9.7	23.1	0.140

All the reference values are for women in their 20s. ^(a)^ For menstruating women. E%, energy percent; EAR, estimated average requirement; AI, adequate intake.

**Table 5:** Qualitative food intake frequency survey results according to snack intake habits; days per week.

Number of Days per Week	Without-Snack-Intake Group	Snack-Intake Group	*p*-Value
	n = 31	n = 39	
Avoiding consuming sweets and soft drinks (e.g., juice)	3.3 ± 2.4	2.8 ± 2.2	0.353
Consuming three meals per day (breakfast, lunch, and dinner)	5.0 ± 2.0	5.6 ± 1.8	0.207
Consuming staple foods (e.g., rice, bread, and noodles)	6.2 ± 1.4	6.5 ± 0.9	0.272
Consuming eggs (or egg dishes)	4.5 ± 2.1	4.8 ± 2.0	0.543
Consuming meat and fish side dishes	4.7 ± 1.9	5.0 ± 1.9	0.535
Consuming soy products (e.g., tofu and natto)	3.7 ± 1.9	4.5 ± 1.8	0.084
Consuming vegetables high in beta-carotene (e.g., carrots, spinach, and broccoli)	3.5 ± 1.8	4.0 ± 2.0	0.274
Consuming light-colored vegetables (e.g., cucumbers, cabbage, and lettuce)	4.7 ± 1.6	5.0 ± 1.4	0.464
Consuming milk/yogurt	3.4 ± 2.4	4.2 ± 2.2	0.145
Consuming fruit (including 100% juice)	2.6 ± 2.0	3.5 ± 2.0	0.072
Consuming breakfast	5.0 ± 2.2	5.5 ± 1.9	0.383
Consuming snacks	0.0 ± 0.0	3.7 ± 1.8	<0.001

The minimum value is 0 and the maximum value is 7 because we inquired about how many days a week the participants consumed or refrained from consuming snacks.

**Table 6:** Types of snacks consumed by female athletes and the number and percentage of participants who consumed snacks.

	n	%
Rice balls	18	36.7
Bread	10	20.4
Energy gels	5	10.2
Protein (supplement)	5	10.2
Bananas	4	8.2
Steamed chicken	3	6.1
100% fruit juice	2	4.1
Other	2	4.1

**Table 7:** Types of dietary supplements consumed by female athletes and the participants’ number of users and percentage of these supplements.

	n	%
Protein	11	42.3
Vitamin complex	5	19.2
Amino acid	4	15.4
Iron	2	7.7
Vitamin C	2	7.7
Carnitine	1	3.8
Bifidobacterium tablets	1	3.8
Total number of responses	26	100.0

%, percentage of participants; multiple answers possible.

## Data Availability

The data that support the findings of this study are available from the corresponding author.
